# Impact of blood culture positivity at intensive care unit admission on mortality in infective endocarditis: Machine learning and deep learning-based causal inference models

**DOI:** 10.1371/journal.pone.0333351

**Published:** 2025-11-06

**Authors:** Min Woo Kang, Shin Young Ahn, Yoonjin Kang

**Affiliations:** 1 Department of Internal Medicine, Korea University Guro Hospital, Seoul, Korea; 2 Department of Thoracic and Cardiovascular Surgery, Seoul National University Hospital, Seoul, Korea; 3 Seoul National University, College of Medicine, Seoul, Korea; Pescara General Hospital, ITALY

## Abstract

**Background:**

Infective endocarditis (IE) carries high in-hospital mortality, particularly among intensive care unit (ICU) patients. The predictive role of blood culture positivity in these patients remains unclear.

**Methods:**

We analyzed 484 adult IE patients from the Medical Information Mart for Intensive Care III (MIMIC-III) database, divided into training (n = 339) and testing (n = 145) cohorts. A suite of demographic, clinical, laboratory, and blood culture variables was used to develop tree-based machine learning models. Random Forest (RF) and Extreme Gradient Boosting (XGB) emerged as top performers and were combined into an ensemble model. SHapley Additive exPlanations (SHAP) quantified variable importance, while the Generative Adversarial Nets for Inference of Individualized Treatment Effects (GANITE) model assessed the average treatment effect (ATE) and conditional treatment effects (CATE) of blood culture positivity on in-hospital mortality across various clinical subgroups.

**Results:**

The ensemble model demonstrated robust performance with an area under the receiver operating characteristic curve (AUROC) of 0.826 and an accuracy of 0.821 on the test set. Blood culture positivity consistently ranked among the top predictors of mortality. SHAP analysis revealed that the presence of bacteremia increased the predicted probability of in-hospital mortality. Specifically, the GANITE model estimated that blood culture positivity raised mortality by 0.9% (95% confidence interval [CI]: −0.9% to 2.6%) in the training set, 7.4% (95% CI: 4.3% to 10.4%) in the test set, and 2.8% (95% CI: 1.2% to 4.4%) overall. Furthermore, CATE analysis highlighted that the adverse impact of blood culture positivity was significantly more pronounced in patients aged 60 years and older, those with systolic blood pressure below 100 mmHg, and in certain endocarditis subtypes.

**Conclusions:**

Blood culture positivity at ICU admission is associated with a modest yet clinically significant increase in in-hospital mortality among IE patients. The application of advanced machine learning and causal inference models enhances risk stratification and may inform more targeted clinical interventions in this high-risk group.

## Introduction

The in-hospital mortality rate of infective endocarditis (IE) remains high, ranging from 13% to 25% [1–5]. IE can result in numerous complications requiring intensive care unit (ICU) care, such as septic shock and cardiogenic shock [6]. Mortality strikingly rises in patients requiring ICU admission, with rates escalating up to 24% to 54% [7]. Therefore, identifying the risk factors for mortality in IE patients, especially those admitted to the ICU, is critically important.

Known risk factors for mortality in IE patients include advanced age, diabetes mellitus, Staphylococcus aureus etiology, embolic events, clinical evolution with heart failure, and septic shock [8,9]. Specifically, for IE patients admitted to the ICU, risk factors for mortality include advanced age, stroke, high Simplified Acute Physiology Score II, Sequential Organ Failure Assessment scores, and Methicillin-resistant Staphylococcus aureus (MRSA) etiology [10,11]. The association between blood culture positivity and mortality in IE patients has been extensively studied, but there is some debate over whether blood culture positivity is a definitive risk factor for mortality [9,12,13]. In infective endocarditis, contemporary registry data (EURO-ENDO) report higher long-term mortality in culture-negative cases compared with culture-positive disease [14]. By contrast, several cohort studies—including a cardiac surgery series and community-acquired IE—reported no significant difference in short- or long-term mortality between culture-negative and culture-positive disease [15,16]. Consistently, a recent systematic review and meta-analysis found no overall mortality difference between culture-positive and culture-negative IE [17]. Taken together, these mixed findings motivate a cautious interpretation of culture positivity as a risk marker and justify our focus on absolute risk differences and sensitivity analyses. However, research on the relationship between blood culture positivity at the time of ICU admission and mortality in ICU-admitted IE patients is limited.

Thus, this study aimed to develop machine learning and deep learning models using the Medical Information Mart for Intensive Care III (MIMIC-III) dataset to predict in-hospital mortality among ICU-admitted endocarditis patients. We attempted to identify the risk factors and important variables in mortality prediction. Additionally, this study adopted a causal inference deep learning model to quantify the increment or decrement of in-hospital mortality based on blood culture positivity at the time of ICU admission.

## Methods

### Ethics statement

This retrospective study using the de-identified MIMIC-III database was approved by the Institutional Review Board of Seoul National University Hospital (IRB No. 2405-061-1535) with a waiver of informed consent.

### Study population

In this study, we utilized the MIMIC-III dataset from Physionet and selected patients with a diagnosis related to endocarditis upon admission from those with ICU admission records. The MIMIC-III database contains deidentified health-related data from over forty thousand patients who stayed in critical care units at the Beth Israel Deaconess Medical Center between 2001 and 2012, offering a comprehensive repository of demographic, clinical, and outcome data [18].

The diagnoses related to endocarditis included a range of conditions such as Syphilitic endocarditis (Syphilitic endocarditis NOS, Syphilitic endocarditis of valve unspecified, Syphilitic endocarditis of mitral valve, Syphilitic endocarditis of aortic valve, Syphilitic endocarditis of tricuspid valve, Syphilitic endocarditis of pulmonary valve), Gonococcal endocarditis, Meningococcal endocarditis, Candida endocarditis, Histoplasmosis endocarditis (Infection by Histoplasma capsulatum endocarditis, Infection by Histoplasma duboisii endocarditis, Histoplasmosis unspecified endocarditis), Coxsackie endocarditis, acute and subacute bacterial endocarditis, acute and subacute infective endocarditis in disease classified elsewhere, acute endocarditis unspecified, endocarditis valve unspecified unspecified cause, endocarditis in disease classified elsewhere, other endocarditis valve unspecified, acute rheumatic endocarditis, and rheumatic disease of the endocardium valve unspecified. We included rheumatic endocarditis and rheumatic disease in this analysis, considering that infective endocarditis can coexist with rheumatic heart disease and that infective endocarditis can sometimes be misdiagnosed as rheumatic endocarditis [19,20]. Only adult patients were included in the analysis, and those with missing vital sign or laboratory data were excluded. For machine learning and deep learning analyses, we randomly divided the entire population into training and testing datasets at a ratio of 7:3.

### Variables for analysis

Demographic data included age and sex. Initial vital signs comprised systolic blood pressure (SBP), diastolic blood pressure (DBP), heart rate (HR), and oxygen saturation (SpO_2_). Laboratory data included white blood cell count (WBC), hemoglobin (Hb), hematocrit (Hct), platelet count (PLT), initial creatinine (initial Cr), baseline creatinine (base Cr), bicarbonate, sodium, and potassium. Laboratory values were taken from the time 24 hours before ICU admission up to 6 hours after admission, with the value measured closest to admission time utilized. Blood culture data included blood culture positivity, Methicillin-sensitive Staphylococcus aureus (MSSA) bacteremia, MRSA bacteremia, Pseudomonas bacteremia, and candidemia. Since it takes at least 48 hours to confirm blood culture positivity [21,22], we collected results from tests performed 48 hours before ICU admission up to 6 hours after admission and used the result closest to the ICU admission time. Additionally, surgery codes were used to determine the presence of open heart surgery, septal repair, annuloplasty, or other heart surgeries. Other vital signs and the norepinephrine rate measured closest to ICU admission time were also included. The occurrence of intubation within 6 hours of admission was also considered as a variable.

For comparing variables between groups with and without in-hospital mortality, t-tests were used for continuous variables, and chi-square tests were used for categorical variables. Multivariable logistic regression analysis was conducted using all variables to assess the association between in-hospital mortality and each variable. A P value <0.05 was considered statistically significant.

### Machine learning and deep learning models predicting in-hospital mortality

The analysis was conducted using the Python PyCaret package, where ten different tree-based algorithms were applied to the training data with 10-fold cross-validation: Extra Trees, CatBoost, Extreme Gradient Boosting (XGB), Random Forest (RF), Light Gradient Boosting Machine, Gradient Boosting, Decision Tree, Ada Boost, Logistic Regression, Ridge, Linear Discriminant Analysis, and Support Vector Machine. During training, we performed 10-fold cross-validation on the training data to select the top two algorithms with the highest average area under the receiver operating characteristic curve (AUROC) values. These two algorithms then underwent hyperparameter tuning with another 10-fold cross-validation. Subsequently, the variable importance for in-hospital mortality prediction was assessed for these two algorithms. The predictive performance of these models was then evaluated on the test data. Additionally, SHapley Additive exPlanations (SHAP) values were calculated for the entire dataset using the top two algorithms, creating summary plots to illustrate the impact of each variable on in-hospital mortality. SHAP values, based on game theory, help to demystify machine learning predictions by quantifying the contribution of each input variable [23].

Evaluation metrics for model performance included accuracy, AUROC, and F1-score. The performance of the models was compared using receiver operating characteristic (ROC) curves and decision curve analysis (DCA) plots. For deep learning, the TabTransformer model was utilized. After training on the training data using the same methodology as in machine learning, model performance was evaluated on the test data.

### Causal inference deep learning model

We used generative adversarial nets for inference of individualized treatment effects (GANITE) as an established causal deep learning framework that (i) imputes counterfactual outcomes via an adversarial generator–discriminator and (ii) then estimates individualized effects with an inference network [24]. We chose GANITE not for methodological novelty but because it is well validated, flexibly accommodates non-linear covariate–treatment interactions and treatment-effect heterogeneity, and yields patient-level probabilistic potential outcomes that map directly to absolute risk differences—a clinically interpretable quantity for our binary mortality endpoint. Compared with representation-balancing or propensity-regularized alternatives, GANITE’s direct counterfactual modeling could align better with our goal of estimating admission-time risk differences under routine ICU data, while maintaining transparency and stability for sensitivity analyses. Blood culture positivity was set as the treatment variable, while in-hospital mortality was designated as the outcome variable. To assess the effectiveness of the model’s training, accuracy, AUROC, and F1-score were evaluated on the test dataset. Additionally, calibration performance was analyzed using calibration plots for the training and test data.

Furthermore, the average treatment effect (ATE) was calculated to determine how mortality changed, on average, when bacteremia was positive compared to when it was negative across the training, test, and total datasets. Conditional treatment effects (CATE) were also assessed based on age (60 years and older vs. younger than 60), intubation within the first 6 hours, the presence of open heart surgery, sex, heart rate (≥100 vs. < 100 beats per minute), SBP (≥100 vs. < 100 mmHg), norepinephrine administration, and endocarditis etiology (bacterial, rheumatic, and candida), as well as the performance of annuloplasty. T-tests were employed to compare CATE between different groups.

To delve deeper into the impact of blood culture positivity on in-hospital mortality, the dataset was split into two groups based on the extent to which blood culture positivity influenced the likelihood of in-hospital mortality: whether the influence of blood culture positivity on the probability of in-hospital mortality was positive or negative. The variables used in the analysis were compared between these two groups. Differences in continuous variables between these groups were analyzed using t-tests, while categorical variables were compared using chi-square tests. Furthermore, a multivariable logistic regression analysis was conducted to examine the odds ratio for whether blood culture positivity increases the probability of in-hospital mortality, thereby identifying which variable characteristics exert a more pronounced effect on the likelihood of blood culture positivity.

### Sensitivity analysis

We performed two prespecified sensitivity analyses. First, to assess generalizability and address potential overfitting, we conducted stratified 5-fold cross-validation (stratified by in-hospital mortality). In each outer fold, the GANITE model was trained on four folds with all hyperparameters fixed to those of the primary analysis, and evaluated on the held-out fold. We also formed out-of-fold (OOF) predictions by concatenating all held-out predictions across folds to report pooled AUROC and pooled ATE. Pooled ATE 95% confidence intervals (CIs) were obtained via nonparametric bootstrap with 500 replicates. Second, because rheumatic endocarditis could confound inference, we excluded patients with rheumatic endocarditis and repeated the same 5-fold cross-validation protocol, using identical covariates, preprocessing, and hyperparameters as in the primary analysis.

## Results

### Study population

**Fig 1:**
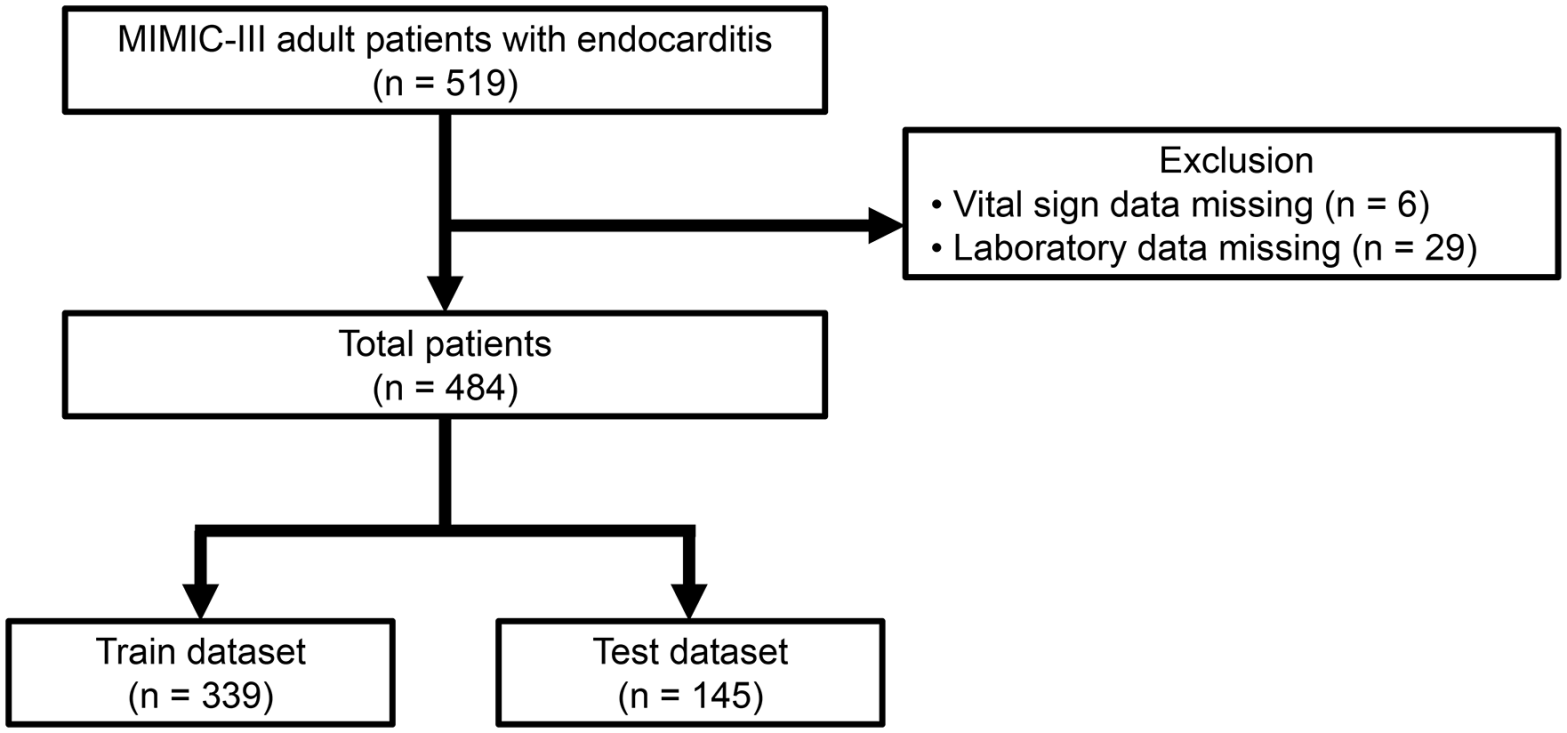
Study population.

Out of a total of 519 endocarditis patients, 484 patients with complete vital sign and laboratory data were included in the analysis, as illustrated in Fig 1. A total of 484 patients were included in the analysis, among which 71 (14.7%) experienced in-hospital mortality. The mean age of the study population was 59.3 years, with the age of patients who died due to endocarditis being significantly higher (mean age 65.1 years) compared to those who survived (mean age 58.2 years) (p = 0.001). The mortality group had significantly lower percentages of males and those who had open heart surgery, but a higher incidence of bacteremia (Table 1). The dataset was randomly divided into 339 training and 145 testing datasets, with no significant differences in nearly all variables between the two groups (S1 Table).

**Table 1:** Baseline characteristics.

Variable	Total population (N = 484)	In-hospital mortality (N = 71)	No in-hospital mortality (N = 413)	P-value
Age (years)	59.25 ± 8.92	65.14 ± 11.08	58.24 ± 8.64	0.001
Male (%)	66% (318)	51% (36)	68% (282)	0.006
SBP (mmHg)	117.18 ± 9.92	112.87 ± 15.02	117.92 ± 11.88	0.147
DBP (mmHg)	60.68 ± 7.62	58.68 ± 10.44	61.02 ± 7.84	0.333
Heart Rate (/min)	93.84 ± 8.67	93.32 ± 13.39	93.93 ± 9.75	0.831
SpO^2^ (%)	96.92 ± 4.58	96.18 ± 7.44	97.04 ± 4.97	0.268
WBC (10^3^/L)	14.18 ± 7.06	17.00 ± 10.61	13.69 ± 7.35	0.003
Hemoglobin (g/dL)	9.97 ± 1.73	10.16 ± 2.15	9.94 ± 1.61	0.340
Hematocrit (%)	30.05 ± 5.25	30.84 ± 7.56	29.91 ± 5.39	0.182
Platelet (10^3^/L)	238.17 ± 68.87	194.92 ± 104.87	245.61 ± 75.58	0.001
Creatinine (mg/dL)	2.18 ± 1.10	2.63 ± 2.04	2.11 ± 1.24	0.061
Base Creatinine (mg/dL)	1.90 ± 1.00	2.23 ± 1.80	1.84 ± 1.08	0.118
Bicarbonate (mmol/L)	23.88 ± 3.75	21.27 ± 5.05	24.33 ± 3.43	<0.001
Sodium (mmol/L)	136.36 ± 3.08	136.52 ± 5.41	136.33 ± 2.94	0.819
Potassium (mmol/L)	4.26 ± 0.73	4.44 ± 1.09	4.23 ± 0.70	0.066
Annuloplasty (%)	1% (4)	0% (0)	1% (4)	0.999
Open Heart Surgery (%)	25% (123)	6% (4)	29% (119)	0.001
Septal Repair (%)	1% (6)	1% (1)	1% (5)	0.999
Other Heart Surgery (%)	1% (6)	0% (0)	1% (6)	0.999
Blood culture positive (%)	28% (136)	39% (28)	26% (108)	0.031
MSSA (%)	5% (24)	6% (4)	5% (20)	>0.999
MRSA (%)	6% (30)	11% (8)	5% (22)	0.099
Pseudomonas (%)	0% (2)	1% (1)	0% (1)	0.679
Candidemia (%)	0% (2)	1% (1)	0% (1)	0.679
Bacterial Endocarditis (%)	90% (436)	86% (61)	91% (375)	0.291
Candida Endocarditis (%)	1% (3)	0% (0)	1% (3)	0.999
Rheumatic Endocarditis (%)	2% (8)	6% (4)	1% (4)	0.019
Endocarditis NOS (%)	7% (36)	7% (5)	8% (31)	>0.999
Intubation within 6 hours (%)	4% (18)	0% (0)	4% (18)	0.999
Norepinephrine (mcg/kg/min)	0.05 ± 0.15	0.10 ± 0.30	0.04 ± 0.13	0.168

Data are presented as mean ± standard deviation for continuous variables and number (%) for categorical variables.

Abbreviation: SBP, systolic blood pressure; DBP, diastolic blood pressure; SpO^2^, oxygen saturation; WBC, white blood cell; MSSA, methicillin-sensitive Staphylococcus aureus; MRSA, methicillin-resistant Staphylococcus aureus; NOS, not otherwise specified.

*Chi-square test for categorical variables and t-test for continuous variables.

### Logistic regression for in-hospital mortality

After adjusting for all variables, the multivariable logistic regression analysis showed that higher age (Odds Ratio [OR]: 1.03 [1.01–1.05], p: 0.010), initial creatinine (OR: 1.70 [1.08–2.69], p: 0.220), and WBC (OR: 1.10 [1.05–1.14], p: < 0.001) were associated with an increased risk of in-hospital mortality. Conversely, lower bicarbonate levels (OR: 0.90 [0.84–0.96], p: 0.001) were linked to a higher mortality risk. Blood culture positive, MRSA bacteremia, and MSSA bacteremia did not show a statistically significant correlation with in-hospital mortality (Table 2).

**Table 2:** Odds ratio for in-hospital mortality.

Variable	Odds Ratio^*^	95% Confidence interval	P-value
Age	1.030	1.007-1.054	0.010
Creatinine	1.703	1.079-2.687	0.022
Base creatinine	0.542	0.326-0.902	0.018
Bicarbonate	0.896	0.838-0.957	0.001
WBC	1.097	1.054-1.141	<0.001
Platelet	0.996	0.994-0.999	0.008
Open heart surgery	0.117	0.034-0.397	0.001
Blood culture positive	1.171	0.518-2.648	0.704
MSSA bacteremia	0.686	0.162-2.898	0.608
MRSA bacteremia	1.181	0.357-3.910	0.785

*Adjusted variables: sex, systolic blood pressure, diastolic blood pressure, oxygen saturation, heart rate, hemoglobin, hematocrit, sodium, potassium, norepinephrine infusion rate, annuloplasty, septal repair, other heart surgery, candida endocarditis, bacterial endocarditis, endocarditis NOS, rheumatic endocarditis, pseudomonas bacteremia, candidemia, intubation within 6 hours of intensive care admission.

Abbreviation: WBC, white blood cell; MSSA, methicillin-sensitive Staphylococcus aureus; MRSA, methicillin-resistant Staphylococcus aureus.

### Machine learning and deep learning models predicting in-hospital mortality

In the 10-fold cross-validation on the training data, the RF and XGB models demonstrated the highest performance. Following hyperparameter tuning, an ensemble model combining these two was developed. The RF, XGB, and ensemble models exhibited AUROCs of 0.821, 0.809, and 0.826, respectively, and accuracies of 0.814, 0.807, and 0.821 in the test data (S2 Table and S1 Fig). The TabTransformer model showed an AUROC of 0.645 and an accuracy of 0.841 on the test data. Decision Curve Analysis (DCA) plots indicated that the RF, XGB, and ensemble models performed similarly well, whereas the TabTransformer showed relatively poorer performance.

**Fig 2:**
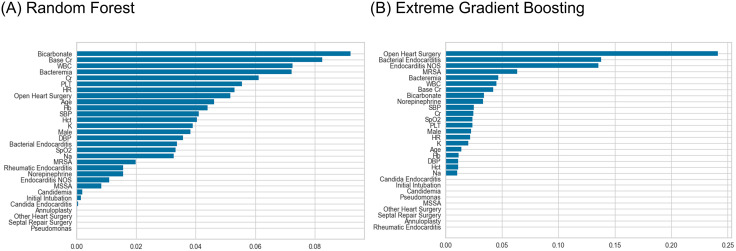
Variable importance of Random Forest and Extreme Gradient Boosting models. Cr, creatinine; WBC, white blood count; PLT, platelet; HR, heart rate; Hb, hemoglobin; SBP, systolic blood pressure; Hct, hematocrit; K, potassium; DBP, diastolic blood pressure; SpO_2_, peripheral oxygen saturation; Na, sodium; MRSA, methicillin-resistant Staphylococcus aureus; NOS, not otherwise specified; MSSA, methicillin-sensitive Staphylococcus aureus.

**Fig 3:**
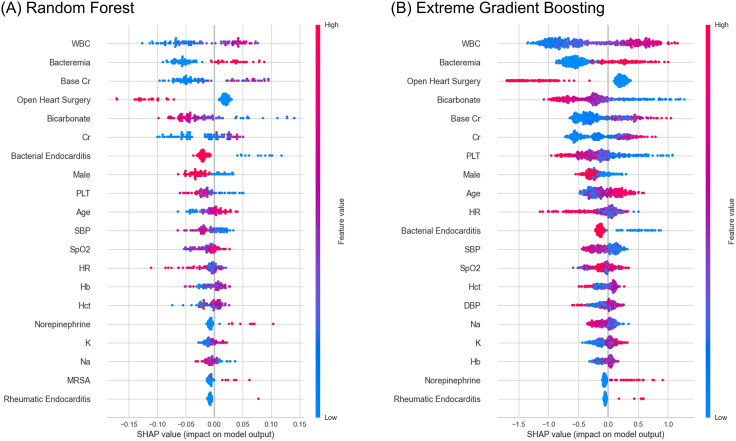
Shapley additive explanations summary and waterfall plot of Random Forest and Extreme Gradient Boosting models. WBC, white blood cell; Cr, creatinine; PLT, platelet; SBP, systolic blood pressure; SpO_2_, peripheral oxygen saturation; HR, heart rate; Hb, hemoglobin; Hct, hematocrit; K, potassium; Na, sodium; MRSA, methicillin-resistant Staphylococcus aureus; DBP, diastolic blood pressure.

The variable importance analysis in the RF and XGB models identified blood culture positivity as the fourth and fifth most critical variable for predicting in-hospital mortality, respectively (Fig 2). The SHAP value plots indicated a tendency for both models to predict in-hospital mortality when bacteremia was positive (Fig 3). Additionally, trends suggested that lower heart rate, lower WBC counts, the performance of open heart surgery, younger age, and lower norepinephrine administration rates were associated with lower risks of in-hospital mortality.

### Causal inference deep learning model

The GANITE model analysis revealed that blood culture positive cases at ICU admission increased the probability of in-hospital mortality by 0.9% (95% [CI]: −0.9–2.6%) in the training data, 7.4% (95% CI: 4.3–10.4%) in the test data, and 2.8% (95% CI: 1.2–4.4%) across the total data (Table 3). The GANITE model’s AUROC was 0.768, with an accuracy of 0.834 in the test data (S2 Fig). In the test data, the GANITE model showed relatively moderate calibration performance but tended to slightly overestimate when predicting high in-hospital mortality.

**Table 3:** Average treatment effect for in-hospital mortality and evaluation indexes of deep learning based causal inference model.

	Train data	Test data	Total data
Accuracy	1.000	0.834	0.950
F1-score	1.000	0.429	0.829
AUC	1.000	0.768	0.944
ATE	0.009 (−0.009-0.026)	0.074 (0.043-0.104)	0.028 (0.012-0.044)

Abbreviation: AUC, area under the receiver operating characteristic; ATE, average treatment effect.

**Fig 4:**
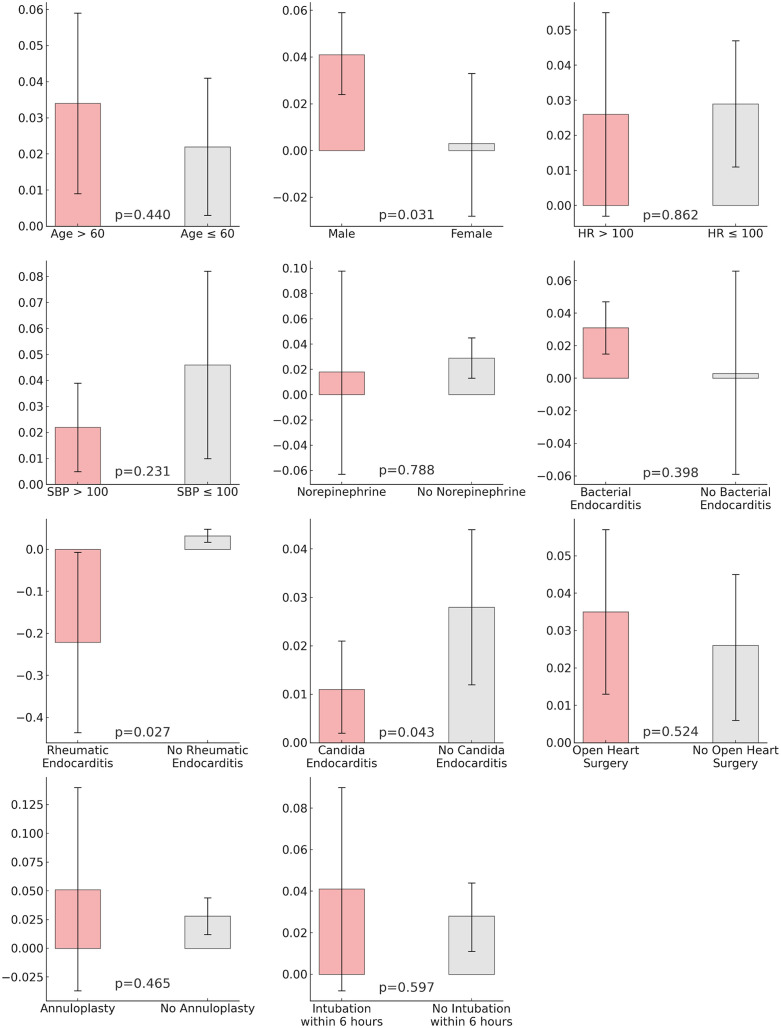
Bar plots of conditional average treatment effect. HR, heart rate; SBP, systolic blood pressure.

The CATE analysis showed that blood culture positivity increased the tendency for in-hospital mortality under all conditions except in subjects with rheumatic endocarditis (Fig 4). However, the 95% CI for the heart rate > 100, norepinephrine, and annuloplasty groups included zero, indicating no significant effect. Statistically significant differences were observed when comparing the CATEs of opposing conditions in males, subjects with rheumatic endocarditis, and Candida endocarditis. Other conditions showed no statistically significant differences.

The median effect of blood culture positivity on in-hospital mortality was 1.57%. When dividing the groups into those where the effect of blood culture positivity was ≥ 1.57% or not, no significant differences were found in variables other than age, SBP, DBP, bicarbonate, creatinine, baseline creatinine, platelet, and potassium, including open heart surgery, annuloplasty, heart rate, WBC count, and norepinephrine administration rate (S3 Table). Only age, SBP, and PLT showed significant correlations in multivariable logistic regression for high blood culture positivity’s impact on in-hospital mortality, which is over 1.57% (Table 4). Older age, lower SBP, and higher PLT were associated with a higher effect of blood culture positivity on in-hospital mortality.

**Table 4:** Odds ratio for high effectiveness of blood culture positive for in-hospital mortality.

	Odds Ratio	95% Confidence interval	P-value
Age	1.043	1.028-1.059	<0.001
SBP	0.971	0.960-0.982	<0.001
DBP	1.003	0.988-1.019	0.670
Platelet	0.998	0.996-1.000	0.031

*Adjusted variables: sex, oxygen saturation, heart rate, white blood cell, hemoglobin, hematocrit, creatinine, base creatinine, bicarbonate, sodium, potassium, norepinephrine infusion rate, open heart surgery, annuloplasty, septal repair, other heart surgery, candida endocarditis, bacterial endocarditis, endocarditis NOS, rheumatic endocarditis, methicillin-sensitive Staphylococcus aureus bacteremia, methicillin-resistant Staphylococcus aureus bacteremia, pseudomonas bacteremia, candidemia, intubation within 6 hours of intensive care admission.

Abbreviation: SBP, systolic blood pressure; DBP, diastolic blood pressure.

### Sensitivity analysis

In 5-fold cross-validation, the mean AUROC across folds was 0.670 ± 0.047, with a pooled OOF AUROC of 0.666. The mean ATE across folds was 4.2% ± 3.0%, and the pooled OOF ATE was 4.2% (95% CI, 2.7%−5.7%) (S4 Table). After excluding rheumatic endocarditis and repeating the analysis, the mean AUROC was 0.709 ± 0.066, with a pooled OOF AUROC of 0.697. The mean ATE across folds was 4.6% ± 1.9%, and the pooled OOF ATE was 4.6% (95% CI, 3.4%−5.8%) (S5 Table).

## Discussion

This study not only developed a machine learning model to predict in-hospital mortality among ICU-admitted endocarditis patients but also explored which variables influence mortality through statistical and machine learning techniques. Specifically, by employing a deep learning causal inference model for the first time, we observed an association between blood culture positivity and a slightly higher in-hospital mortality under similar patient conditions. Importantly, these patterns remained robust in prespecified sensitivity analyses, including patient-level stratified 5-fold cross-validation and a cohort restricted to non-rheumatic endocarditis. Our results showed that the ensemble model achieved the highest AUROC of 0.821 in the test dataset, suggesting that the mortality prediction model itself could aid clinical practice.

We investigated the impact of various factors on in-hospital mortality using logistic regression—a classical statistical model well-known for its ability to elucidate the associations between clinical variables and outcomes. However, logistic regression has limitations, such as assessing the model’s goodness of fit. Thus, we supplemented this with an analysis of SHAP values from tree-based machine learning models, offering additional clinical insights into the relationship between variables and mortality. Both logistic regression and machine learning models indicated that patients not undergoing open heart surgery tended to have a lower risk of in-hospital mortality. This observation aligns with previous studies [3,9].

Although blood culture positivity did not show significant results in logistic regression, machine learning models revealed its tendency to influence mortality. This discrepancy led us to employ a deep learning causal inference model to assess the association and estimate potential effects of bacteremia on in-hospital mortality. Since randomization is not possible for the presence of blood culture positivity, using a deep learning causal inference model to explore potential causal relationships under standard assumptions is very useful in evaluating the impact of bacteremia on clinical outcomes. In this study, we leveraged the causal inference deep learning framework to elucidate the impact of blood culture positivity on mortality rates among ICU patients with endocarditis. Unlike traditional statistical methods, which often struggle with direct assessments and may overestimate effects in certain sample sizes, the causal inference deep learning model provides numerical estimates of how blood culture positivity may be associated with mortality risk. This nuanced analysis yields a more intuitive interpretation of results, differentiating it markedly from classical statistical approaches [25]. Beyond merely conducting subgroup analyses or calculating CATE, the causal inference deep learning model adeptly segregates patient populations based on their predicted mortality risk depending on blood culture positivity. It identifies characteristics of patients for whom blood culture positivity may be associated with a higher mortality risk, thereby offering clearer insights into patient vulnerability.

The presence of persistent blood culture positivity, particularly blood culture positivity at ICU admission, influencing patient survival outcomes has yet to be definitively established. Whether blood culture positivity is a definitive risk factor for mortality remains a subject of debate [13]. However, there is previous research indicating that persistent bacteremia serves as a risk factor for mortality [26]. The presence of blood culture positivity at the time of ICU admission suggests a high bloodstream pathogen burden and a potential state of severe sepsis. For this reason, the presence of blood culture positivity at ICU admission could be thought to increase the risk of mortality. However, it is challenging to assume that blood culture-negative endocarditis has a lower mortality risk due to a lower bloodstream pathogen burden, given the heterogeneous nature of bacteremia-negative endocarditis patients, including those with rheumatic endocarditis [27]. Furthermore, some endocarditis cases may be caused by specific pathogens not detectable by blood culture, leading to variations in mortality among blood culture-negative endocarditis depending on the etiology [27]. Therefore, our study results suggest that blood culture positivity was associated with a higher mortality risk, the magnitude of this increase was not substantial.

Old age and lower SBP subjects appeared to have higher effects of blood culture positivity on in-hospital mortality. The mortality due to severe sepsis in elderly patients is 1.3–1.5 times higher than that in younger cohorts [28,29]. Therefore, blood culture positivity at ICU admission in older patients is likely to predict higher mortality. The tendency for lower SBP to be associated with higher mortality when blood culture is positive may reflect the high mortality observed in septic shock scenarios where blood pressure drops and blood cultures are positive [30].

Several limitations are acknowledged in this research. First, although the total number of subjects was 484—which is substantial for an ICU endocarditis cohort—it remains modest for machine and deep learning analyses. In addition, extreme sparsity in several subgroups limits statistical power for both model training and heterogeneity analyses, increasing variance, heightening the risk of overfitting, and reducing generalizability. Obtaining large-scale data under specific conditions like ICU admissions for endocarditis is challenging. Second, the absence of variables related to heart function, such as vegetation size and ejection fraction, due to the constraints of the MIMIC-III dataset, prevented their inclusion. Incorporating these variables would have significantly reduced the analysis pool due to missing data. Third, we observed a discrepancy in ATE CIs between the development-split training and testing sets, likely reflecting overfitting and sampling variability in a modest-sized cohort. Our out-of-fold, patient-level stratified 5-fold cross-validation mitigated this issue and yielded more stable—albeit modest—effects; nevertheless, the small magnitudes warrant cautious interpretation and external validation. Fourth, the overall cohort size and extreme sparsity in several subgroups limit statistical power for both model training and heterogeneity analyses, increasing variance and the risk of overfitting and reducing generalizability. Fourth, key infective endocarditis prognostic variables—most notably echocardiographic features such as vegetation size, ejection fraction, and valve involvement—were unavailable in MIMIC-III. As a result, residual confounding may persist despite adjustment for available clinical.

In conclusion, our study suggests that the presence of blood culture positivity at ICU admission in ICU-admitted endocarditis patients was associated with a small increase in-hospital mortality compared to its absence. This finding, supported by advanced machine learning and causal inference analyses, highlights specific patient characteristics associated with higher estimated risk when blood culture positivity is present.

## Supporting information

S1 Table Baseline characteristics of train and test sets. (DOCX)

S2 Table Model performance for in-hospital mortality prediction. (DOCX)

S3 Table Comparison by treatment-effect strata. (DOCX)

S4 Table Stratified 5-fold cross-validation results. (DOCX)

S5 Table Cross-validation excluding rheumatic endocarditis. (DOCX)

S1 Fig ROC and decision curve analysis of prediction models. (DOCX)

S2 Fig ROC and calibration of the causal inference model. (DOCX)

## References

[1] Alagna L, Park LP, Nicholson BP, Keiger AJ, Strahilevitz J, Morris A, et al. Repeat endocarditis: analysis of risk factors based on the International Collaboration on Endocarditis - Prospective Cohort Study. Clin Microbiol Infect. 2014;20(6):566–75. doi: 10.1111/1469-0691.12395 24102907

[2] Bor DH, Woolhandler S, Nardin R, Brusch J, Himmelstein DU. Infective endocarditis in the U.S., 1998-2009: a nationwide study. PLoS One. 2013;8(3):e60033. doi: 10.1371/journal.pone.0060033 23527296 PMC3603929

[3] Muñoz P, Kestler M, De Alarcon A, Miro JM, Bermejo J, Rodríguez-Abella H, et al. Current Epidemiology and Outcome of Infective Endocarditis: A Multicenter, Prospective, Cohort Study. Medicine (Baltimore). 2015;94(43):e1816. doi: 10.1097/MD.0000000000001816 26512582 PMC4985396

[4] Murdoch DR, Corey GR, Hoen B, Miró JM, Fowler VG Jr, Bayer AS, et al. Clinical presentation, etiology, and outcome of infective endocarditis in the 21st century: the International Collaboration on Endocarditis-Prospective Cohort Study. Arch Intern Med. 2009;169(5):463–73. doi: 10.1001/archinternmed.2008.603 19273776 PMC3625651

[5] Slipczuk L, Codolosa JN, Davila CD, Romero-Corral A, Yun J, Pressman GS, et al. Infective endocarditis epidemiology over five decades: a systematic review. PLoS One. 2013;8(12):e82665. doi: 10.1371/journal.pone.0082665 24349331 PMC3857279

[6] Pericàs JM, Hernández-Meneses M, Muñoz P, Martínez-Sellés M, Álvarez-Uria A, de Alarcón A, et al. Characteristics and Outcome of Acute Heart Failure in Infective Endocarditis: Focus on Cardiogenic Shock. Clin Infect Dis. 2021;73(5):765–74. doi: 10.1093/cid/ciab098 33560404

[7] Cresti A, Baratta P, De Sensi F, Aloia E, Sposato B, Limbruno U. Clinical Features and Mortality Rate of Infective Endocarditis in Intensive Care Unit: A Large-Scale Study and Literature Review. Anatol J Cardiol. 2024;28(1):44–54. doi: 10.14744/AnatolJCardiol.2023.3463 38167795 PMC10796247

[8] Chu VH, Cabell CH, Benjamin DK Jr, Kuniholm EF, Fowler VG Jr, Engemann J, et al. Early predictors of in-hospital death in infective endocarditis. Circulation. 2004;109(14):1745–9. doi: 10.1161/01.CIR.0000124719.61827.7F 15037538

[9] Marques A, Cruz I, Caldeira D, Alegria S, Gomes AC, Broa AL, et al. Risk Factors for In-Hospital Mortality in Infective Endocarditis. Arq Bras Cardiol. 2020;114(1):1–8. doi: 10.36660/abc.20180194 31751437 PMC7025303

[10] Joffre J, Dumas G, Aegerter P, Dubée V, Bigé N, Preda G, et al. Epidemiology of infective endocarditis in French intensive care units over the 1997-2014 period-from CUB-Réa Network. Crit Care. 2019;23(1):143. doi: 10.1186/s13054-019-2387-8 31027489 PMC6485099

[11] Leroy O, Georges H, Devos P, Bitton S, De Sa N, Dedrie C, et al. Infective endocarditis requiring ICU admission: epidemiology and prognosis. Ann Intensive Care. 2015;5(1):45. doi: 10.1186/s13613-015-0091-7 26621197 PMC4666184

[12] Meidrops K, Zuravlova A, Osipovs JD, Kalejs M, Groma V, Petrosina E, et al. Comparison of outcome between blood culture positive and negative infective endocarditis patients undergoing cardiac surgery. J Cardiothorac Surg. 2021;16(1):147. doi: 10.1186/s13019-021-01532-9 34044847 PMC8161995

[13] Suardi LR, de Alarcón A, García MV, Ciezar AP, Hidalgo Tenorio C, Martinez-Marcos FJ, et al. Blood culture-negative infective endocarditis: a worse outcome? Results from a large multicentre retrospective Spanish cohort study. Infect Dis (Lond). 2021;53(10):755–63. doi: 10.1080/23744235.2021.1925342 34038316

[14] Kong WKF, Salsano A, Giacobbe DR, Popescu BA, Laroche C, Duval X, et al. Outcomes of culture-negative vs. culture-positive infective endocarditis: the ESC-EORP EURO-ENDO registry. Eur Heart J. 2022;43(29):2770–80. doi: 10.1093/eurheartj/ehac307 35695691 PMC9459867

[15] Meidrops K, Zuravlova A, Osipovs JD, Kalejs M, Groma V, Petrosina E, et al. Comparison of outcome between blood culture positive and negative infective endocarditis patients undergoing cardiac surgery. J Cardiothorac Surg. 2021;16(1):147. doi: 10.1186/s13019-021-01532-9 34044847 PMC8161995

[16] Siciliano RF, Mansur AJ, Castelli JB, Arias V, Grinberg M, Levison ME, et al. Community-acquired culture-negative endocarditis: clinical characteristics and risk factors for mortality. Int J Infect Dis. 2014;25:191–5. doi: 10.1016/j.ijid.2014.05.005 24971520

[17] Rehman H, Rawat A, Gill FS, Kukreja A, Oliinyk Y, Chaudhari SS, et al. Clinical Outcomes in Blood Culture-Positive Versus Blood Culture-Negative Infective Endocarditis: A Systematic Review and Meta-Analysis. Cureus. 2025;17(7):e88134. doi: 10.7759/cureus.88134 40821345 PMC12357163

[18] Johnson AEW, Pollard TJ, Shen L, Lehman L-WH, Feng M, Ghassemi M, et al. MIMIC-III, a freely accessible critical care database. Sci Data. 2016;3:160035. doi: 10.1038/sdata.2016.35 27219127 PMC4878278

[19] Baskerville CA, Hanrahan BB, Burke AJ, Holwell AJ, Rémond MGW, Maguire GP. Infective endocarditis and rheumatic heart disease in the north of Australia. Heart Lung Circ. 2012;21(1):36–41. doi: 10.1016/j.hlc.2011.08.010 21924682

[20] Hajsadeghi S, Hassanzadeh M, Hajahmadi M, Kadivar M. Concurrent diagnosis of infective endocarditis and acute rheumatic fever: A case report. J Cardiol Cases. 2018;17(5):147–50. doi: 10.1016/j.jccase.2017.12.011 30279878 PMC6149577

[21] Pardo J, Klinker KP, Borgert SJ, Trikha G, Rand KH, Ramphal R. Time to positivity of blood cultures supports antibiotic de-escalation at 48 hours. Ann Pharmacother. 2014;48(1):33–40. doi: 10.1177/1060028013511229 24259644

[22] Pollack LA, Srinivasan A. Core elements of hospital antibiotic stewardship programs from the Centers for Disease Control and Prevention. Clin Infect Dis. 2014;59 Suppl 3(Suppl 3):S97–100. doi: 10.1093/cid/ciu542 25261548 PMC6521960

[23] Lundberg SM, Lee SI. A unified approach to interpreting model predictions. In: Proceedings of the 31st International Conference on Neural Information Processing Systems, Long Beach, California, USA, 2017. 4768–77.

[24] Yoon J, Jordon J, Schaar Mvd, editors. GANITE: Estimation of Individualized Treatment Effects using Generative Adversarial Nets. International Conference on Learning Representations; 2018.

[25] Smit JM, Krijthe JH, Kant WMR, Labrecque JA, Komorowski M, Gommers DAMPJ, et al. Causal inference using observational intensive care unit data: a scoping review and recommendations for future practice. NPJ Digit Med. 2023;6(1):221. doi: 10.1038/s41746-023-00961-1 38012221 PMC10682453

[26] López J, Sevilla T, Vilacosta I, Sarriá C, Revilla A, Ortiz C, et al. Prognostic role of persistent positive blood cultures after initiation of antibiotic therapy in left-sided infective endocarditis. Eur Heart J. 2013;34(23):1749–54. doi: 10.1093/eurheartj/ehs379 23144047

[27] McHugh J, Saleh OA. Updates in Culture-Negative Endocarditis. Pathogens. 2023;12(8):1027. doi: 10.3390/pathogens12081027 37623987 PMC10459830

[28] Martin GS, Mannino DM, Moss M. The effect of age on the development and outcome of adult sepsis. Crit Care Med. 2006;34(1):15–21. doi: 10.1097/01.ccm.0000194535.82812.ba 16374151

[29] Nasa P, Juneja D, Singh O, Dang R, Arora V. Severe sepsis and its impact on outcome in elderly and very elderly patients admitted in intensive care unit. J Intensive Care Med. 2012;27(3):179–83. doi: 10.1177/0885066610397116 21436163

[30] Lal A, Rayes H, O’Horo JC, Singh TD, Gajic O, Kashyap R. Septic shock definitions and associated outcomes in blood culture positive critically ill patients. Ann Transl Med. 2023;11(5):192. doi: 10.21037/atm-22-5147 37007579 PMC10061476

