# Supplementary material for: Impact of blood culture positivity at intensive care unit admission on mortality in infective endocarditis: Machine learning and deep learning-based causal inference models
Source: PLoS One. 2025 Nov 6;20(11):e0333351. doi: 10.1371/journal.pone.0333351 (PMC12591472; doi:10.1371/journal.pone.0333351)
Supplement: S1 Table — (DOCX) [file pone.0333351.s001.docx]

Supplementary Table S1. Baseline characteristics of train and test data set

| Variable | Total data  (N=484) | Train data  (N=339) | Test data  (N=145) | P-value |
| --- | --- | --- | --- | --- |
| Age (years) | 59.25 ± 16.84 | 58.32 ± 17.19 | 61.43 ± 15.67 | 0.052 |
| Male (%) | 65.7 (318) | 65.2 (221) | 66.9 (97) | 0.797 |
| SBP (mmHg) | 117.18 ± 23.46 | 118.52 ± 24.89 | 114.03 ± 19.78 | 0.035 |
| DBP (mmHg) | 60.68 ± 16.39 | 60.54 ± 16.91 | 61.01 ± 15.11 | 0.758 |
| Heart Rate (/min) | 93.84 ± 20.20 | 93.78 ± 20.85 | 93.98 ± 18.92 | 0.917 |
| SpO^2^ (%) | 96.92 ± 5.16 | 96.88 ± 5.73 | 97.01 ± 3.62 | 0.769 |
| WBC (10^3^/L) | 14.18 ± 7.75 | 14.39 ± 7.89 | 13.67 ± 7.50 | 0.340 |
| Hemoglobin (g/dL) | 9.97 ± 1.91 | 9.97 ± 1.88 | 9.96 ± 1.97 | 0.955 |
| Hematocrit (%) | 30.05 ± 5.39 | 30.02 ± 5.45 | 30.11 ± 5.47 | 0.865 |
| Platelet (10^3^/L) | 238.17 ± 151.08 | 240.19 ± 156.03 | 233.46 ± 140.20 | 0.638 |
| Creatinine (mg/dL) | 2.18 ± 2.47 | 2.14 ± 2.25 | 2.28 ± 2.83 | 0.594 |
| Base Creatinine (mg/dL) | 1.90 ± 2.13 | 1.87 ± 2.07 | 1.95 ± 2.46 | 0.734 |
| Bicarbonate (mmol/L) | 23.88 ± 4.83 | 23.91 ± 4.79 | 23.81 ± 4.85 | 0.826 |
| Sodium (mmol/L) | 136.36 ± 5.39 | 136.39 ± 5.73 | 136.30 ± 4.48 | 0.848 |
| Potassium (mmol/L) | 4.26 ± 0.79 | 4.26 ± 0.75 | 4.28 ± 0.68 | 0.729 |
| Annuloplasty (%) | 0.8 (4) | 0.9 (3) | 0.7 (1) | >0.999 |
| Open Heart Surgery (%) | 25.4 (123) | 26.5 (90) | 22.8 (33) | 0.445 |
| Septal Repair (%) | 1.2 (6) | 0.9 (3) | 2.1 (3) | 0.529 |
| Other Heart Surgery (%) | 1.2 (6) | 0.9 (3) | 2.1 (3) | 0.529 |
| Blood culture positive (%) | 28.1 (136) | 28.9 (98) | 26.2 (38) | 0.620 |
| MSSA (%) | 5.0 (24) | 4.4 (15) | 6.2 (9) | 0.549 |
| MRSA (%) | 6.2 (30) | 6.2 (21) | 6.2 (9) | >0.999 |
| Pseudomonas (%) | 0.4 (2) | 0.6 (2) | 0.0 (0) | <0.001 |
| Candidemia (%) | 0.4 (2) | 0.6 (2) | 0.0 (0) | <0.001 |
| Bacterial Endocarditis (%) | 90.1 (436) | 90.9 (308) | 88.3 (128) | 0.482 |
| Candida Endocarditis (%) | 0.6 (3) | 0.6 (2) | 0.7 (1) | >0.999 |
| Rheumatic Endocarditis (%) | 1.7 (8) | 1.8 (6) | 1.4 (2) | >0.999 |
| Endocarditis NOS (%) | 7.4 (36) | 6.8 (23) | 9.0 (13) | 0.517 |
| Intubation within 6 hours (%) | 3.7 (18) | 3.8 (13) | 3.4 (5) | >0.999 |
| Norepinephrine (mcg/kg/min) | 0.05 ± 0.22 | 0.04 ± 0.28 | 0.05 ± 0.25 | 0.798 |
| In-hospital mortality (%) | 14.7 (71) | 14.5 (49) | 15.2 (22) | 0.949 |

Data are presented as mean ± standard deviation for continuous variables and number (%) for categorical variables.

Abbreviation: SBP, systolic blood pressure; DBP, diastolic blood pressure; SpO^2^, oxygen saturation; WBC, white blood cell; MSSA, methicillin-sensitive Staphylococcus aureus infections; MRSA, methicillin-resistant Staphylococcus aureus; NOS, not otherwise specified.

*Chi-square test for categorical variables and t-test for continuous variables.
