# Supplementary material for: Impact of blood culture positivity at intensive care unit admission on mortality in infective endocarditis: Machine learning and deep learning-based causal inference models
Source: PLoS One. 2025 Nov 6;20(11):e0333351. doi: 10.1371/journal.pone.0333351 (PMC12591472; doi:10.1371/journal.pone.0333351)
Supplement: S2 Table — (DOCX) [file pone.0333351.s002.docx]

Supplementary Table S2. Performance of models predicting In-hospital mortality

|  | Train AUC | Train Accuracy | Train F1-score | Train Recall | Test AUC | Test Accuracy | Test F1-score | Test Recall |
| --- | --- | --- | --- | --- | --- | --- | --- | --- |
| Ensemble | 0.971 | 0.944 | 0.796 | 0.755 | 0.826 | 0.821 | 0.35 | 0.318 |
| Random Forest | 0.973 | 0.947 | 0.809 | 0.776 | 0.821 | 0.814 | 0.308 | 0.273 |
| XGBoost | 0.951 | 0.947 | 0.804 | 0.755 | 0.809 | 0.807 | 0.333 | 0.318 |
| TabTransformer | 0.834 | 0.861 | 0.113 | 0.061 | 0.645 | 0.841 | 0 | 0 |

Abbreviation: AUC, area under the receiver operating characteristic; XGBoost, Extreme Gradient Boosting.
