# Supplementary material for: Impact of blood culture positivity at intensive care unit admission on mortality in infective endocarditis: Machine learning and deep learning-based causal inference models
Source: PLoS One. 2025 Nov 6;20(11):e0333351. doi: 10.1371/journal.pone.0333351 (PMC12591472; doi:10.1371/journal.pone.0333351)
Supplement: S3 Table — (DOCX) [file pone.0333351.s003.docx]

Supplementary Table S3. Comparing lower and higher effect than median value group in total population

| Variable | Treatment effect < median^*^ | Treatment effect ≥ median^*^ | P-value^†^ |
| --- | --- | --- | --- |
| Age (years) | 54.85 ± 3.56 | 63.65 ± 3.01 | <0.001 |
| Male (%) | 61.6 (149) | 69.8 (169) | 0.069 |
| SBP (mmHg) | 122.96 ± 5.19 | 111.39 ± 4.04 | <0.001 |
| DBP (mmHg) | 62.95 ± 3.51 | 58.41 ± 3.10 | 0.002 |
| Heart Rate (/min) | 95.13 ± 4.49 | 92.55 ± 3.71 | 0.159 |
| SpO^2^ (%) | 96.93 ± 0.87 | 96.91 ± 1.22 | 0.958 |
| WBC (10^3^/L) | 13.68 ± 1.68 | 14.67 ± 1.47 | 0.159 |
| Hemoglobin (g/dL) | 9.92 ± 0.39 | 10.02 ± 0.39 | 0.573 |
| Hematocrit (%) | 29.84 ± 1.10 | 30.25 ± 1.12 | 0.418 |
| Platelet (10^3^/L) | 258.17 ± 37.40 | 218.18 ± 21.59 | 0.003 |
| Creatinine (mg/dL) | 1.93 ± 0.41 | 2.44 ± 0.57 | 0.020 |
| Base Creatinine (mg/dL) | 1.67 ± 0.37 | 2.13 ± 0.50 | 0.019 |
| Bicarbonate (mmol/L) | 24.66 ± 0.92 | 23.10 ± 0.99 | <0.001 |
| Sodium (mmol/L) | 136.63 ± 1.13 | 136.09 ± 1.06 | 0.276 |
| Potassium (mmol/L) | 4.18 ± 0.16 | 4.34 ± 0.15 | 0.019 |
| Annuloplasty (%) | 0.4 (1) | 1.2 (3) | 0.616 |
| Open Heart Surgery (%) | 27.3 (66) | 23.6 (57) | 0.404 |
| Septal Repair (%) | 0.8 (2) | 1.7 (4) | 0.681 |
| Other Heart Surgery (%) | 2.1 (5) | 0.4 (1) | 0.218 |
| MSSA (%) | 2.9 (7) | 7.0 (17) | 0.060 |
| MRSA (%) | 4.1 (10) | 8.3 (20) | 0.090 |
| Pseudomonas (%) | 0.4 (1) | 0.4 (1) | >0.999 |
| Candidemia (%) | 0.4 (1) | 0.4 (1) | >0.999 |
| Bacterial Endocarditis (%) | 90.1 (218) | 90.1 (218) | >0.999 |
| Candida Endocarditis (%) | 1.2 (3) | 0.0 (0) | 0.248 |
| Rheumatic Endocarditis (%) | 2.9 (7) | 0.4 (1) | 0.075 |
| Endocarditis NOS (%) | 5.4 (13) | 9.5 (23) | 0.119 |
| Intubation within 6 hours (%) | 4.1 (10) | 3.3 (8) | 0.810 |
| Norepinephrine (mcg/kg/min) | 0.04 ± 0.06 | 0.05 ± 0.05 | 0.900 |

Data are presented as mean ± standard deviation for continuous variables and number (%) for categorical variables.

Abbreviation: SBP, systolic blood pressure; DBP, diastolic blood pressure; SpO^2^, oxygen saturation; WBC, white blood cell; MSSA, methicillin-sensitive Staphylococcus aureus infections; MRSA, methicillin-resistant Staphylococcus aureus; NOS, not otherwise specified.

*Median treatment effect: 0.0157

†Chi-square test for categorical variables and t-test for continuous variables.
