# Supplementary material for: Impact of blood culture positivity at intensive care unit admission on mortality in infective endocarditis: Machine learning and deep learning-based causal inference models
Source: PLoS One. 2025 Nov 6;20(11):e0333351. doi: 10.1371/journal.pone.0333351 (PMC12591472; doi:10.1371/journal.pone.0333351)
Supplement: S4 Table — (DOCX) [file pone.0333351.s004.docx]

Supplementary Table S4. Results of stratified 5-fold cross-validation

| Metric | Estimate |
| --- | --- |
| AUROC |  |
| 5-fold mean ± standard deviation | 0.670 ± 0.047 |
| Pooled out-of-fold | 0.666 |
| ATE |  |
| 5-fold mean ± standard deviation | 4.2% ± 3.0% |
| Pooled out-of-fold (95% confidence interval) | 4.2% (2.7% - 5.7%) |
