# Supplementary material for: Impact of blood culture positivity at intensive care unit admission on mortality in infective endocarditis: Machine learning and deep learning-based causal inference models
Source: PLoS One. 2025 Nov 6;20(11):e0333351. doi: 10.1371/journal.pone.0333351 (PMC12591472; doi:10.1371/journal.pone.0333351)

Supplementary Figure S1. Receiver operating characteristic curve and decision curve analysis plots of prediction models. XGBoost, Extreme Gradient Boosting


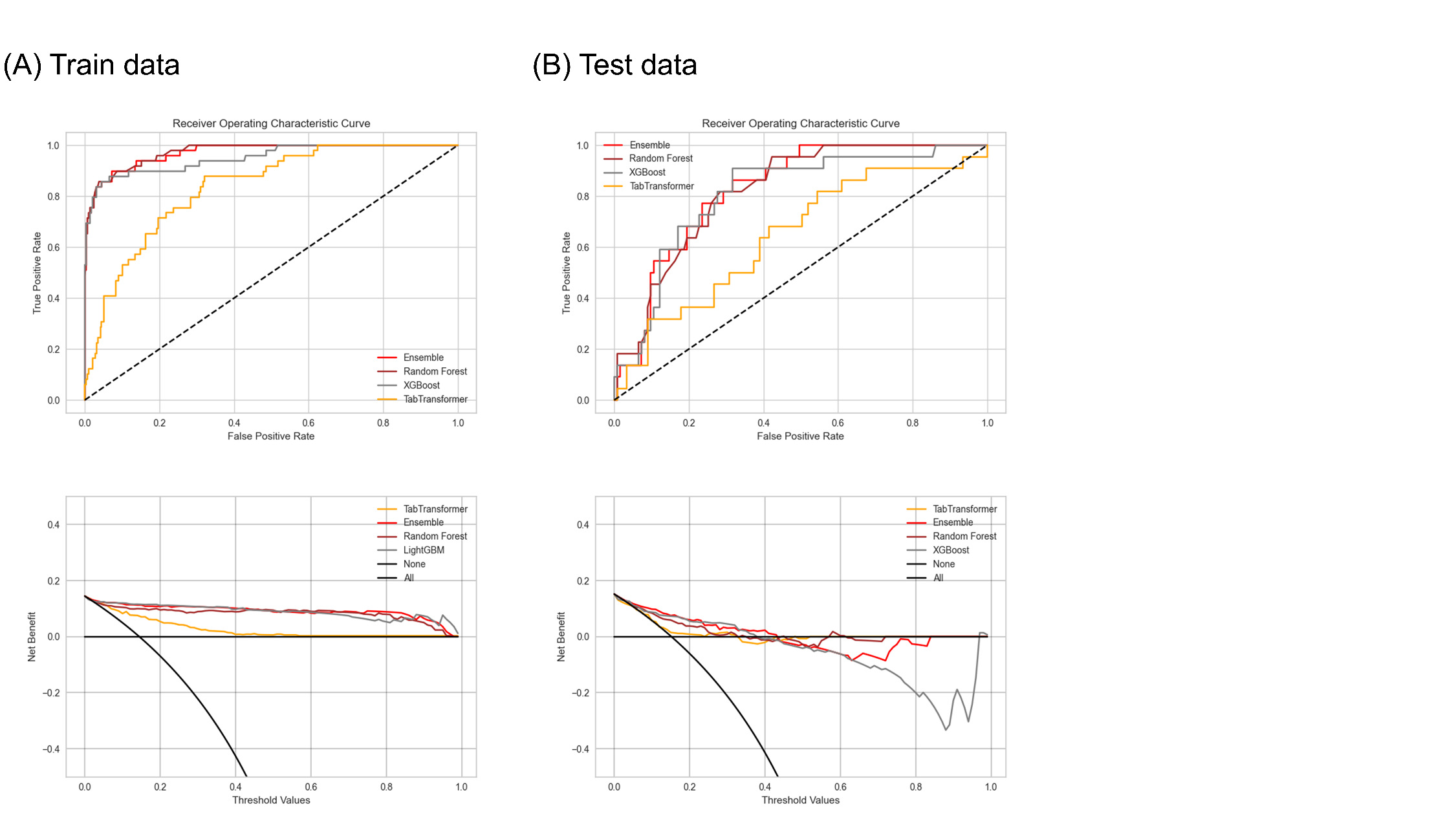

Supplement: S1 Fig — (DOCX) [file pone.0333351.s006.docx]
