# Supplementary material for: Impact of blood culture positivity at intensive care unit admission on mortality in infective endocarditis: Machine learning and deep learning-based causal inference models
Source: PLoS One. 2025 Nov 6;20(11):e0333351. doi: 10.1371/journal.pone.0333351 (PMC12591472; doi:10.1371/journal.pone.0333351)

Supplementary Figure S2. Receiver operating characteristic curve and calibration plot of deep learning based causal inference model


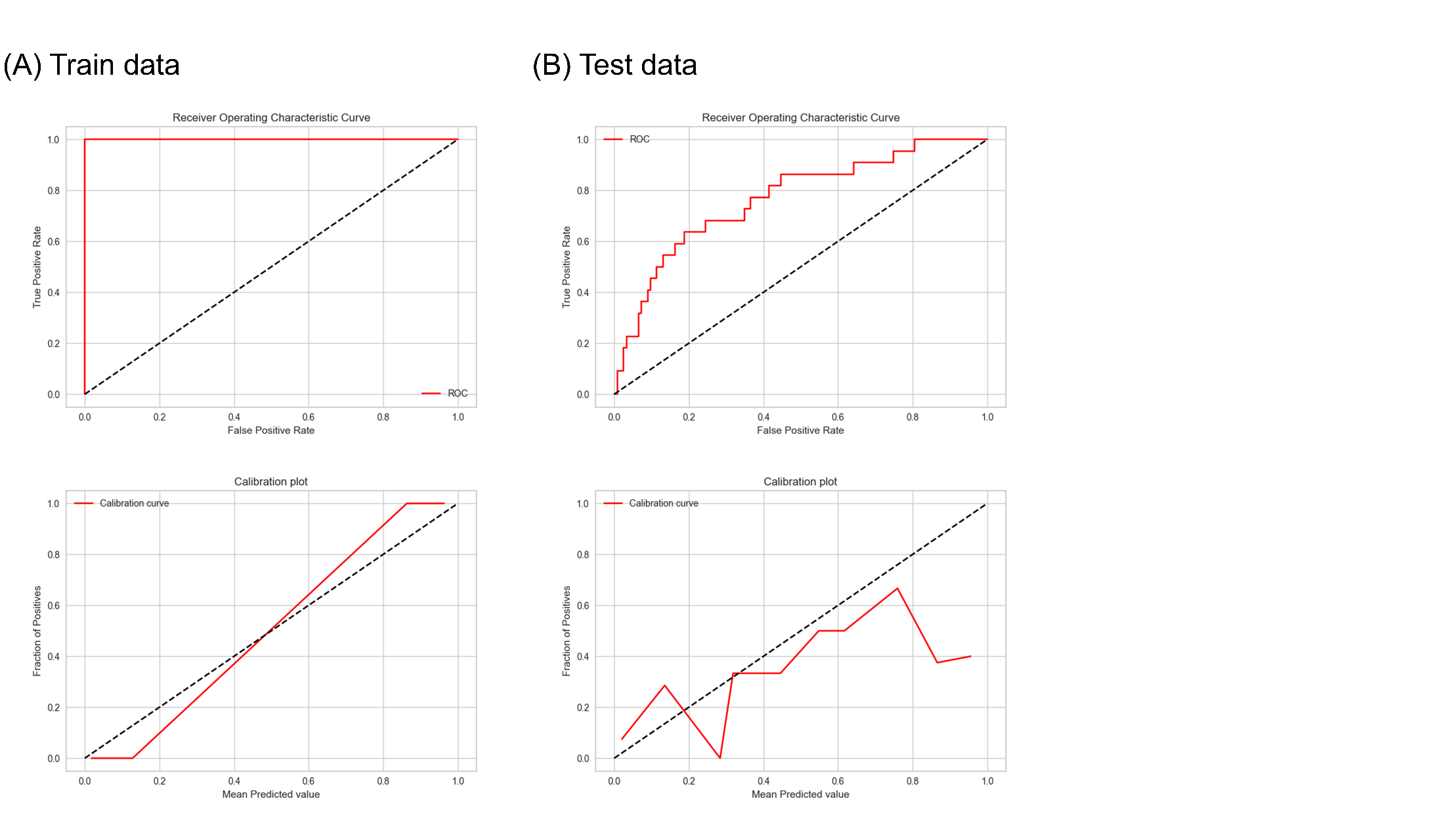

Supplement: S2 Fig — (DOCX) [file pone.0333351.s007.docx]
